# Evidence-Informed Approach to De-Prescribing of Atypical Antipsychotics (AAP) in the Management of Behavioral Expressions (BE) in Advanced Neurocognitive Disorders (NCD): Results of a Retrospective Study

**DOI:** 10.1192/j.eurpsy.2024.187

**Published:** 2024-08-27

**Authors:** A. S. Luthra, R. LinBin Gao, P. Carducci, J. Sue, S. Remers

**Affiliations:** ^1^Department of Psychiatry and Behavioural Neurosciences, McMaster University; ^2^Department of Pharmacy, St. Peter’s Hospital, Hamilton; ^3^School of Pharmacy, University of Waterloo, Waterloo; ^4^Clinical Neuropsychologist, St. Peter’s Hospital, Hamilton; ^5^Homewood Health Inc, Guelph, Canada

## Abstract

**Introduction:**

Diagnosis of behaviors in advanced neurocognitive disorders (aNCD) is one of exclusion, and the framework has been laid out in DSM-V. However, clinical assessments in aNCD become increasingly unreliable, and commonly used psychometric tools for clinical assessments lack reliability and validity, thereby making outcomes unreliable. Consequently, the syndromic and symptom management approaches for behaviors in aNCD behaviors have yielded poor results. To address this, the focus has shifted towards understanding the ‘meaning’ of behaviors in aNCD, recognizing them as a ‘mode of communication’. To date, there are no existing frameworks to ascribe ‘meaning’ to behaviors in aNCD.

**Objectives:**

LuBAIR™ paradigm is the first step in offering such a framework for understanding the ‘purpose’ and ‘meaning’ of behaviors in NCD. The ‘meaning’ ascribed to each behavioral category was used to guide the use of atypical antipsychotics in their management. De-prescribing was attempted on patients who qualified to enter this retrospective study. De-prescribing was defined as successful if individuals were completely withdrawn from AAP and remained off them for 60 days without the re-emergence of behaviors.

**Methods:**

The data collected on the second occasion, in the successful and failed de-prescribed groups, were compared in this retrospective study. MANOVA, Chi-Square paired *t*-test statistical analyses were used to detect the differences in the behavioral categories between the two cohorts. Cohen d was used to measure effect size.

**Results:**

Patients who did not have Mis-Identification and Goal-Directed Expressions were more likely to successfully de-prescribe: X2 (1, *N* = 40) = 29.119 *p* < 0.0001 and X2 (1, *N* = 40) = 32.374, *p* < 0.0001, respectively. Alternatively, the same behavioral categories were more likely present in patients who failed de-prescribing: MANOVA and paired *t*-test (*p* < 0.0001). Atypical antipsychotics, in their role as an antipsychotic and mood stabilizer, may be used to manage Mis-Identification and Goal-Directed Expressions, respectively.

**Conclusions:**

LuBAIR paradigm has the potential to guide the development of specific behavioral care plans and the use of AAP in managing individual behavioral categories. AAP use can be justified for managing Misidentification and Goal-Directed Expressions. Vocal expressions may warrant the use of AAP, pending further study. The LuBAIR paradigm offers guidance for de-prescribing AAP for all other behavioral categories in the LuBAIR Inventory. This study is also a preliminary step in validating the psychological theories used to support the individual categories. This workshop will educate the participants on the LuBAIR paradigm and its application in developing person-centered interventions for behaviors in a NCD.

**Disclosure of Interest:**

None Declared

